# A Qualitative Study of National Perspectives on Advancing Social Prescribing Using Co‐Design in Canada

**DOI:** 10.1111/hex.14144

**Published:** 2024-07-10

**Authors:** Marianne Saragosa, Kate Mulligan, Sonia Hsiung, Srija Biswas, Kiffer Card, Paul C. Hébert, Vivian Welch, Michelle L. A. Nelson

**Affiliations:** ^1^ Science of Care Institute, Lunenfeld‐Tanenbaum Research Institute Sinai Health Toronto Ontario Canada; ^2^ Institute of Health Policy, Evaluation and Management University of Toronto Toronto Ontario Canada; ^3^ Dalla Lana School of Public Health University of Toronto Toronto Ontario Canada; ^4^ Canadian Institute for Social Prescribing Canadian Red Cross Toronto Ontario Canada; ^5^ Faculty of Health Sciences Simon Fraser University Vancouver British Columbia Canada; ^6^ Bruyère Research Institute University of Ottawa Ottawa Ontario Canada

**Keywords:** co‐design, qualitative, social prescribing

## Abstract

**Introduction:**

Social prescribing offers a formal pathway of connecting patients in the health system with sources of support within the community to help improve their health and well‐being. Since its launch in March 2022, the Canadian Institute for Social Prescribing has acted as a collective impact network to identify, connect and build upon established social prescribing initiatives using a co‐design methodology. The institute received input from a participant advisory council, co‐design partners and several communities of interest groups. This study aimed to describe the perceptions of the Canadian Institute for Social Prescribing's role in advancing social prescribing using a co‐design approach and the barriers and facilitators to implementing social prescribing in Canada.

**Methods:**

We used a qualitative descriptive study design, document analysis, participant observation and semi‐structured individual interviews (*n* = 7) with members of the Canadian Institute for Social Prescribing co‐design group and the institute's leadership. We also analysed documents, field notes and transcripts using codebook thematic analysis.

**Results:**

Four themes were developed representing the facilitators of implementing the Canadian Institute for Social Prescribing to support social prescribing: *Creating relational mechanisms* (i.e., partnerships and connections), *Bringing awareness to social prescribing and contributing to the evidence* (i.e., values and beliefs), *Addressing systemic conditions* (i.e., having a common language for social prescribing and organizing the community health sector) and *Enabling funding and policy to drive social prescribing initiatives* (i.e., shifting evidence into policy and securing sustainable funding).

**Conclusion:**

Participants' reflections on the co‐design process demonstrated that the Canadian Institute for Social Prescribing development provided networking opportunities and shared resources relevant to social prescribing. Co‐design efforts also fostered relational and informational support, which laid the necessary groundwork in Canada to overcome the complex interplay between the macro‐ and micro‐level settings in which social prescribing is practiced.

**Patient or Public Contribution:**

The interviews and observations involved participants with lived experience of delivering, receiving or advocating for social prescribing.

## 1 Introduction

There have been repeated calls for intersectoral collaboration to address the persistence of health inequities [1], which largely depend on the social determinants of health. Intersectoral collaboration is a strategy to improve population health through partners across other sectors recognizing their work's impact on health, breaking down barriers and building new partnerships to promote health equity and sustainability [2]. As a mechanism of intersectoral collaboration, co‐creation might have a strong and lasting impact on health and broader outcomes in the local and national settings where health inequities can be tackled [3]. Co‐creation aligns with participatory action, engaging and enabling stakeholders in co‐design to construct a shared understanding that facilitates collective action, creating useful solutions [4]. Evidence suggests that co‐design effectively engages stakeholders in developing and implementing social prescribing programmes within a community setting [5].

Social prescribing, the prescribing of non‐medical, community or social activities, originated in the United Kingdom and has begun to take hold. Husk et al. [6]. It is gaining traction in other parts of the world [7], including Canada. The practice links individuals to non‐clinical services within the local community with entry points, including primary care, social services or self‐referral, by leveraging existing health and social systems to address various psychosocial factors [8]. With an emerging evidence base, numerous global examples of advocacy for social prescribing exist in developed countries [9, 10], involving targeted efforts towards prioritized populations (e.g., older adults and newcomers) and engaging with hyper‐local contexts and available resources [8]. For system leaders wanting to pursue social prescribing, countries were suggested to develop cross‐national leadership networks committed to advancing social prescribing [8]. In Canada, such leaders have taken steps to do just that by creating a pan‐Canadian social prescribing institute, which has provided an opportunity to understand how to scale innovations nationally.

Social prescribing has been adopted in Canada predominantly through localized initiatives [8] and piloted social prescribing pathways [11]. Positive outcomes for patients and volunteers in the social prescription programme [11] paved the way for additional social prescribing initiatives in Canada [12]. This qualitative descriptive study is part of a more extensive research programme focused on the Canadian Institute for Social Prescribing (hereafter referred to as ‘the institute’), launched in 2022 to build on these and other diverse social prescribing initiatives nationwide. As a collective pan‐Canadian social prescribing network, the institute identified, connected and built on social prescribing initiatives using a co‐design methodology [12]. This study examines the experiences and perceptions of those co‐designing the national platform for systematizing community and grassroots social prescribing initiatives and the key challenges and facilitators.

The inherent complexity of national implementation efforts is well described [13]. Therefore, considering how the pan‐Canadian scaling of social prescribing was perceived and experienced by those involved can inform our understanding of the determinants of national scale‐up of interventions situated at the meso‐ and micro‐level nexus. Our qualitative descriptive study aimed to understand national perspectives within the newly formed Canadian Institute for Social Prescribing by answering the following research questions: (1) What were the perceptions of the institute's role in advancing social prescribing using a co‐design approach? and (2) What were the barriers and facilitators to implementing social prescribing locally and nationally?

## 2 Materials and Methods

### 2.1 Study Design and Theoretical Orientation

This qualitative descriptive study was part of a series of evaluation streams addressing different priorities on the effectiveness, acceptability, feasibility and relevance of social prescribing in Canada. Our study was underpinned by pragmatism and the sense that knowledge is based on experience, which is socially shared [14] and based on collaborative efforts [15]. Pragmatism is also grounded in the philosophical roots of co‐design, whereby perception, experience [15] and collaborative, intersectoral engagement are key elements of co‐design [4].

### 2.2 Setting

The institute was launched in March 2022 with funding from the Public Health Agency of Canada (PHAC) under the backbone support of the Canadian Red Cross, officially introduced as Canada's ‘hub for connection, collaboration, and community partnerships’ [16]. In the beginning, the institute's leadership team virtually convened representatives of various community‐based organizations, practitioners and academics who expressed interest in the social prescribing movement in Canada to participate in the co‐design process. In addition to the co‐design activity, these individuals were invited to participate in communities of interest supporting social prescribing by discussing the practice in the scope of clinical practice, policy, research and evidence, knowledge mobilization and communications. The institute also convened a Participant Advisory Council of individuals with lived experience participating as recipients in social prescribing. Between February 2022 and January 2024, the institute co‐designed social prescribing principles, practices and implementation strategies and a concept map to support social prescribing in Canada with representatives from community agencies, clinicians and policy leaders, the participant advisory council and researchers [17, 18].

**Figure 1:**
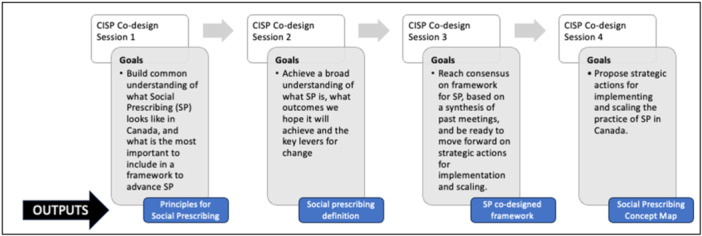
Overview of the institute's co‐design approach to creating a theory of change. Each workshop builds upon the knowledge gained in the previous one.

The Double Diamond framework guided the co‐design process, formed by two diamonds representing exploring an issue more widely or deeply (divergent thinking) and then taking focused action (convergent thinking) [19]. We observed four virtual co‐design sessions between September 2022 and May 2023. According to the initial agenda, the convening aimed to create a framework to advance social prescribing. Two members from the Tamarack Institute and the core institute leadership team facilitated the sessions. Specific goals were outlined for each (see Figure 1), and as a result, specific outputs were created.

### 2.3 Participants and Recruitment

For the semi‐structured interviews, study authors (M.S. and M.L.A.N.) worked with the institute leadership to determine the sampling strategy drawing from various institute stakeholder groups. Recruitment was purposeful based on preselected criteria [20] consisting of participants self‐identifying as members or employees of an approved organization of the institute's development process and involved in at least one facet of the institute's development (i.e., co‐design, communities of interest and/or institute leadership role). The institute leadership distributed a standardized invitation to participate email to an available listserv in January 2023, which allowed those interested in participating to indicate their interest through email. Each potential participant received an informed consent form through email and provided written consent before the interview. This study received ethics approval from the Sinai Health Research Ethics Board (REB#22‐0189‐E). Participant observations included the following institute‐related activities: co‐design planning meetings, co‐design sessions and community interest meetings by institute leadership team members, representatives from community‐based health and social care agencies and government, arts and culture, clinicians and academics. A waiver of consent was received for the observations; however, participants received an approved information sheet about the study.

### 2.4 Data Collection

The study authors used semi‐structured interviews, participation observation, field notes and document analysis to answer the research questions.

#### 2.4.1 Semi‐Structured Interviews

The lead author (M.S.), a female PhD‐prepared registered nurse and postdoctoral fellow with qualitative expertise, conducted one‐time individual virtual semi‐structured interviews to explore participants' experiences and thoughts about participating in the institute's development. Participants were aware of the interviewer from their presence at co‐design sessions; however, relationships were not established before the study commencement. An interview guide was constructed featuring topics on ‘experience in social prescribing,’ ‘readiness for social prescribing implementation’ and ‘expectations and participation’. All interviews were audio‐recorded and transcribed verbatim.

#### 2.4.2 Participation Observations and Field Notes

We (M.S. and M.L.A.N.) conducted participant observations by taking electronic field notes using an REB‐approved template (see Appendix S1) that was developed to capture the underlying purpose behind convening, the event's timing, specific actions that participants were doing, expressions of feelings and desires and reflections or memos by the researcher. A dual but overt role was assumed as the researchers were also engaged in the planning discussions. The observations provided insight into critical decision points, emerging insights and evolving processes within the institute's development.

#### 2.4.3 Document Analysis

The final form of data collection was document analysis. We collected programme material created in the design and development of the institute, such as meeting agendas, meeting summaries and guiding documents (e.g., terms of reference, etc.).

### 2.5 Data Analysis

Documents, field notes and interview transcripts were analysed using codebook thematic analysis [21]. Using this approach, one coder (M.S.) read and coded using inductively derived codes to form a coding framework. M.S. used this framework to code the remaining data using Microsoft Word 365 for data management and engaged in peer debriefing with the senior author (M.L.A.N.) for data consensus.

## 3 Findings

From June 2022 to September 2023, we observed four co‐design and two communities of interest virtual meetings. Attendance ranged between 25 and 43 individuals representing nine Canadian provinces. We also observed seven planning meetings with the consultant team members and the institute leadership members, with attendance ranging between five and seven people, including the evaluators (M.S. and M.L.A.N.).

We collected 28 documents. The documents comprised 11 field notes of official meetings, seven email communications to the co‐design and communities of interest members and 11 reports or data and information briefings, such as survey and research results, terms of reference and social prescribing principles. Interviews were conducted between January 2023 and April 2023 with seven participants (two leaders and five representatives of community‐based organizations). The interviews ranged from 31 to 75 min, with a mean duration of 50 min. One person indicated interest, but did not respond to follow‐up requests to schedule an interview. Transcripts were not returned to participants.

**Figure 2:**
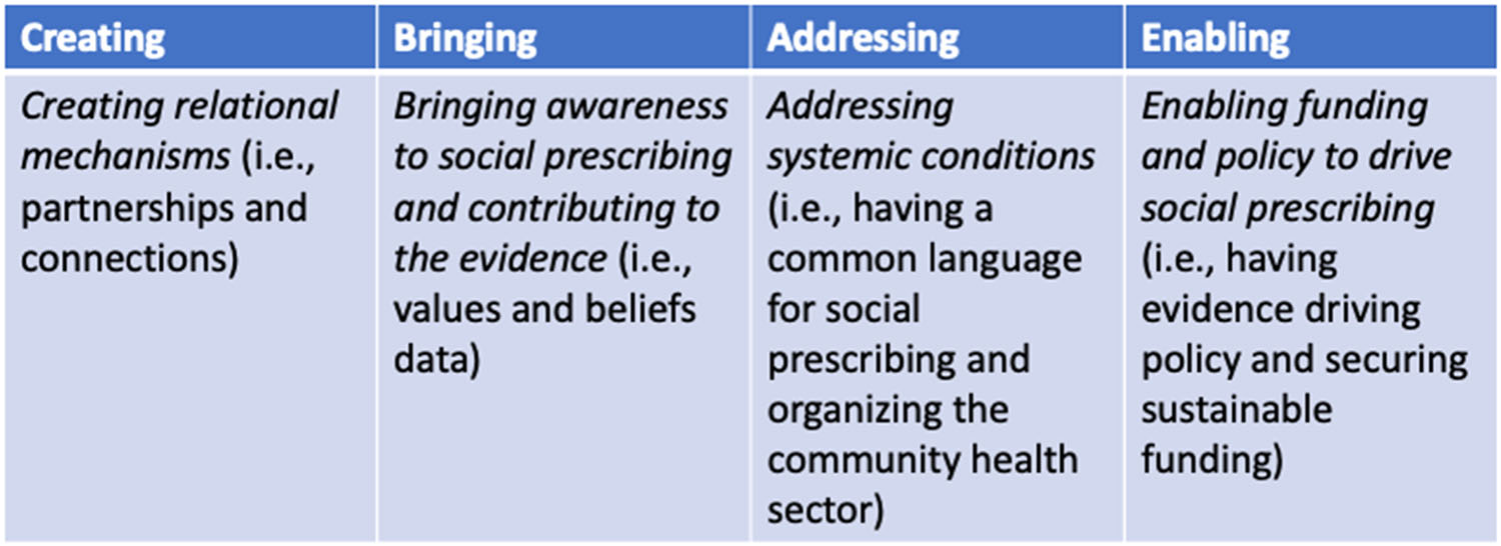
The four themes developed highlight the role of co‐design and the institute in advancing social prescribing in Canada.

We developed four overarching themes representing the experience of convening a pan‐Canadian national stakeholder group for co‐design purposes within the newly formed institute: *Creating relational mechanisms* (i.e., partnerships and connections), *Bringing awareness to social prescribing and contributing to the evidence* (i.e., values and beliefs), *Addressing systemic conditions* (i.e., standardizing social prescribing and organizing the community health sector) and *Enabling funding and policy to drive social prescribing* (see Figure 2).

### 3.1 Forming Partnerships and Building Connections

All participants acknowledged the importance of partnerships and connections in building the institute and implementing social prescribing. The institute, being a new initiative, was viewed to rely on relational mechanisms as it grew and gained ‘legitimacy in larger circles’, according to a participant (P002). The institute's documents also reflected the different engagement methods, including networking (i.e., ‘working groups and communities of practice’) and participating in the co‐design process [22]. However, for others, having established relationships with influential funding organizations (e.g., Ministry of Health, not‐for‐profit) has enabled them to previously test different community‐oriented approaches to caring for more marginalized populations, including social prescribing. Nevertheless, the institute's documents readily included a ‘co‐design partners contact list’ and ways to stay connected beyond scheduled co‐design meetings, for example, on social media platforms like their LinkedIn page.

Participants perceived the attraction and encouragement of participation in social prescribing implementation differently. For those actively involved in social prescribing, there was a strong desire to disseminate resources to reduce ‘reinventing the wheel’ (P001) either provincially or nationally. This sentiment was also noted in the observational data. Attendees at the communities of interest meetings acknowledged concerns about ‘pilot projects’ occurring in some communities and not others. Participants at the communities of interest meetings also mentioned developing ‘collective sets of tools, resources’ as a potential facilitator for implementing social prescribing more broadly. As a mechanism for forming connections, one interview participant mentioned a virtual social prescribing platform and how the institute will be ‘embedded’ in this resource:

Anyone interested in SP can join. It's how we're building our membership to our community of practice because we'll be able to see all the members who are in there, and we'll have their contact details, we can ask questions, we can have discussions within that, we can have. There's an events calendar, where you can see all the upcoming meetings you could talk about, like the work of CISP [the institute], which will all be embedded in that core.

(P005)

Participants also reported their interest in learning how other co‐design participants support social prescribing implementation at their organizations. One participant said that they were developing social prescribing material and curriculum in parallel with learning from the institute and other members:

We're doing all kinds of background capacity‐building stuff. That's where it ties into the institute [CISP]. So, working on a provincial evaluation framework for social prescribing, communication, and material development for healthcare providers, we're also building out a curriculum for the link worker role that we're utilizing in our model. And so, all that stuff around, like best practices and learning and getting information about other projects in other provinces, feels like our interest in the institute lies with wanting to learn from others.

(P002)

In contrast, some expressed worry with the engagement process of the institute co‐design members, given that these individuals were at different stages of the social prescribing implementation process, ‘And I think we assume that everyone is just coming to the table [co‐design] and being interested. And that's not true. People are at very different stages’ (P006). Observational data from the co‐design meetings reinforced varying understandings of social prescribing, particularly around the definition. Attendees raised concerns about over‐medicalizing the approach with language sensitized to the social context. To address this tension and maintain engagement in the innovation, the institute leadership and the co‐design members agreed on a theory of change that was ‘good enough’ to move forward on the strategic roadmap to advancing and scaling social prescribing in Canada.

### 3.2 Bringing Awareness to Social Prescribing and Contributing to the Evidence

Several participants acknowledged the role of the institute in helping legitimize social prescribing, especially within government and non‐conventional spaces (i.e., art galleries and museums), as noted in the following quote:

People just now understand what social prescribing is. And so, we are just building that general awareness in the public sphere, as well. And CISP [the institute] can also help with those awareness campaigns over the next few years.

(P002)

Some participants acknowledged that their organization's culture either enabled or discouraged the priority given to social prescribing. For example, one institution's mandate is to ‘bring art and people together in creative and experimental ways’, which meant that the leadership was not thinking about social prescribing, ‘it's not even on their radar, it's not even important to them’ (P001). The opposite was true for another individual who described their institution as ‘something about our culture or workflow or maturity with using social prescribing or our worldview that is showing some benefit in this approach’ (P004). In turn, for P004, social prescribing is a high priority and drives their involvement in the institute and the co‐design process, ‘I'm participating in the co‐design sessions, but our real interest is in seeing if there are opportunities to test social prescribing’ (P004).

Other participants attending the co‐design meetings looked to the institute for more direction and tangible support with implementing social prescribing. This was evident in the observed feedback about the theory of change framework when attendees inquired how the framework would help bring social prescribing into practice for them.

Most participants mentioned the evidence base for social prescribing as needed to ‘prove the importance of the program’ (P001). The institute's involvement was considered from an ‘academic standpoint’, whereby the institute was evolving the national evidence of social prescribing. Similarly, many also referred to measuring the impact of social prescribing and needing these data and research to ‘talk to governments to make policy change’ (P005). The value of the institute was noted to be in curating the evidence behind social prescribing to demonstrate its impact:

Evaluation projects are demonstrating that social prescribing is feasible and viable, [then it] is likely to be accepted, and will likely have an impact… these community organizations will see it as meaningful and have integrity.

(P007)

The institute supported the implementation process of social prescribing by conducting evaluation projects with academic partners in parallel with the institute's development. One person considered the parallel development and evaluation work as a ‘dance’ that could have benefited the co‐design earlier, ‘While we were trying to define, there were just several pieces that would have been helpful as a foundation to say, this is what we already know’ (P006). Nevertheless, in all the co‐design meetings, attendees mentioned evidence supporting communication about social prescribing and ensuring that it is presented as ‘user‐friendly.’ Access to ‘high‐quality’ social prescribing evidence was mentioned through the co‐design and communities of interest sessions, mainly to understand the work, demonstrate impact and align strategies; however, a barrier reported was ‘data sharing’ to support social prescribing programming. The institute's documentation strongly focused on curating social prescribing evidence noted in an Evidence and Gap Map [23] and preliminary results of understanding the social prescribing needs of Canadians over 55 years [24].

### 3.3 Addressing Systemic Conditions

Interviewed participants and those attending co‐design meetings mentioned how the institute could help change local conditions to better support social prescribing by creating pathways and a common language. Several participants also reported that the institute could help provide a process or an organizing framework for the community health sector. A participant interviewed viewed social prescribing as an organizing framework for the community health sector: ‘… I kept coming along, kept coming, bumping into social prescribing as something that could help to organize the community health sector’ (P007).

Lastly, one participant pointed out that the current primary care system is unsustainable. The institute could help alleviate primary care practitioners' burden by offering a supportive network. For example, they remarked,

But awareness and process are missing. So that's what we're hoping this work will do. Right? Yes, help us to build awareness in the health system, and help us have a process or a system or infrastructure to not add another burden to primary care in terms of trying to, you know, be the system navigator for the patient because they're already carrying everything in the health system that isn't done anywhere else, which is unsustainable.

(P004)

Addressing systemic conditions was also reflected in the institute's documentation as the ‘Key Levers’ needed to change systems and policies. Several participants reported on the design of social prescribing and how the institute informed the adoption of the innovation in the Canadian context. For example, two participants spoke about consistency and standardization of the approach. They viewed the institute as building a ‘coordinated, consistent approach, especially across the country’ (P002). However, some also identified gaps in the innovation focused on sustainability. These people expressed this as having a consistent link worker rather than a ‘revolving door of people’ (P001) and having them integrated into the practice setting, ‘Like it's not going to work if it's just an unknown disintegrated person coming in every once in a while, right’ (P004).

It was essential to be able to adapt the social prescribing innovation; however, to do so, data were suggested to support any changes,

I want to know that if I'm putting in the effort and putting these resources towards developing this program, we're noticing an impact and what that impact is. I can also tweak the program to ensure that it supports positive outcomes.

(P001)

Therefore, some interview participants needed a more relaxed definition of social prescribing to be more adaptable, especially in crises such as the pandemic. During this time, one social prescribing programme had to ‘pivot and change’ to meet the demands of older adult service users,

I'm a huge advocate that we've been doing social prescribing for year, we've just now put a name to it … So I don't think they should have to have a name, eventually we'll get to a place where we don't have to name it anymore. It will just be so foundational and wraparound, [as well as] the services that.

(P005)

According to one person, tension remains in systematizing relational work,

The whole point is that we must systematize [social prescribing] this but keep it human. So, the interesting and maybe fundamental tension for social prescribing is systematizing what has been based on very organic relationships.

(P007)

### 3.4 Enabling Funding and Policy to Drive Social Prescribing

Within the co‐design meetings and interviews, participants mentioned that having sufficient and ongoing funding was vital to the sustainability of social prescribing. While many have benefited from private donations to fund social prescribing projects, there was an emphasis on ongoing government funding. According to P002, in one jurisdiction that received funding from a private foundation, the government now has ‘its fingers on it [social prescribing]’ by conducting its evidence review as a first step towards funding social prescribing on a larger scale. Some talked about funding for a well‐paid ‘Link Worker’ role to avoid turnover and programme disruption:

That's what happens as soon as that person I've been working with, and this happened with NAME Community Health Center. For instance, I developed a program with a woman named NAME. She left that position, and now that program no longer exists with that community health center.

(P001)

For another participant, a limited‐term provincial grant was reported to be vital in advancing social prescribing and partnerships that enabled social prescribing to grow beyond the ‘community health center space’ (P007). However, a dominant theme in the institute documentation was the lack of sustainable funding, acknowledged as a condition that makes the process challenging. Relatedly, one participant strongly suggested that social prescribing be formalized into policy and adopted by the federal or provincial government, ensuring that not only funding is secured but also buy‐in or sign‐off is required by system leaders.

## 4 Discussion

Using thematic analysis, our findings comprised four themes representative of the facilitators and barriers to implementing the institute to support social prescribing.

A robust mechanism needed to support the successful co‐design of social prescribing principles, practices and models was perceived and observed to be relational. Partnerships and connections (i.e., finding the right people to connect with) served two functions: acquiring funding and support to test social prescribing initiatives and, through the institute, convening people who can then potentially establish partnerships. According to implementation science literature, cultivating diverse connections supports developing cross‐sectoral partnerships [25, 26]. For example, Huang et al. [25] describe specific strategies (e.g., listening, developing trust, shared decision‐making, etc.) to be vital at the initial partnering stage to generate buy‐in and mutual goal development. We also found that the engagement of individuals in the implementation process of social prescribing was varied. Those at the more advanced stages desired collective tools and resources to support implementation. At the same time, other members of the co‐design process needed more time to be ready or resourced to implement social prescribing. Notably, the engagement of champions has encouraged the adoption and spread of social prescribing initiatives [27, 28]. Champions may facilitate getting the necessary ‘buy‐in’ to advance social prescribing in Canada. Similarly, Liang et al. observed that engaging formally appointed internal implementation leaders positively impacted the intervention implementation in cancer research [29].

Our findings demonstrated that specific barriers to the collaborative process were external, such as the paucity of data for social prescribing or social prescriptions and the need for government and a common language for social prescribing. However, participants acknowledged that the institute and related co‐design activities, including efforts to curate quality evidence on social prescribing, were considered to legitimize social prescribing to positively affect its perceived value and priority. These findings are consistent with a study of clinicians who had uncertainty about the prevention of dementia. Therefore, study participants perceived a lack of evidence to support dementia risk reduction, undermining the relative importance of promoting brain health [30]. However, providing service providers with evidence for social prescribing may not be enough to support implementation efforts. Many consider the institute reputable and acknowledged its efforts in strategically advancing social prescribing. However, implementers' underlying organizational realities, including resources, capacity, attitudes and culture, may need to be examined. Our study findings also focused on the quality of social prescribing evidence required to support implementation. Extensive work has documented that evidence‐based practices are insufficient to change behaviour, even if the evidence demonstrates an effect on health outcomes [31, 32]. Instead, promising practices must address community needs, be implemented according to the intervention standards to maintain fidelity and be integrated into regular practices for optimal sustainability [33].

In our study, the perspective expressed in the interview and our review of documents demonstrated that the local context and individuals' organizational context were essential to social prescribing implementation. In this way, the institute's collaborative efforts through the co‐design process were described as a vehicle for creating pathways or an organizing framework to support social prescribing. To accelerate the adoption of social prescribing, ensuring knowledge about ‘what works’ in specific contexts is needed when considering the inner and outer settings. Much evidence has appeared in social prescribing implementation in realist evaluations and reviews [34, 35]. Prior research has documented many conditions (mechanisms) and context to identify relevant meso‐ and macro‐level factors (i.e., organizational, social, and policy) that shape social prescribing delivery. For example, previous studies call on the availability of voluntary and community services, compatible institutional ethos, stable funding and monitoring and a new or existing social prescribing workforce of paid staff and volunteers [8, 36, 37]. The findings also showed that assessing systemic conditions required alignment between grassroots organizing and national‐level implementation efforts. That is, while the institute was co‐designing a coordinated approach to social prescribing, there was also mention of adaptability and not being limited by the definition of social prescribing. This is labelled as a ‘triad’ between local implementation sites, supportive stakeholders and robust governmental policies to grow social prescribing at a national scale [8]. In one example from Spain, co‐creating a nature‐based social prescribing programme with citizens, relevant stakeholders and the research sector led to an intervention meant to address loneliness [38].

Perspectives expressed by participants also highlighted the external challenges that individuals and institutions face: financial pressures, competitive funding models and the absence of supportive policies. A significant barrier to implementation is direct funding for the services/activities to support the formal care pathway. Other funding concerns have been raised in the literature, including upfront costs and the workload required to implement social prescribing properly [39]. Since social prescribing has a long history in the United Kingdom, some evidence of financing mechanisms and cost‐effectiveness has emerged [40]. In their systematic review, Dronina et al. [40] found that social prescribing delivered cost savings to the health sector over and above operative costs; however, social prescriber implementers must take caution because the data quality was found in technical evaluation reports. Thus, as more jurisdictions like Canada adopt social prescribing interventions, future research and studies can provide more robust and complete evidence about the cost‐effectiveness of social prescribing.

### 4.1 Strengths and Limitations

This study represents an understanding of the facilitators and barriers to convening national stakeholders for co‐design purposes within the newly formed Canadian Institute for Social Prescribing. We acknowledge the limitations of a small sample of self‐selected participants who participated in an interview and our findings not being provided to participants for feedback, which may have reduced our understanding of additional insights had more people participated or reviewed the findings. We are also aware of the limitation of linking co‐design experience to outcomes. As such, future research considerations include mapping participants' perceptions of the process to outcomes associated with their involvement in co‐design activities. Lastly, as a strength, the observations and field notes corroborated much of what was shared. Our application of multiple data collection methods contributes to the richness of the findings.

## 5 Conclusion

Our findings comprised four themes representative of the perceptions of the institute's role in advancing social prescribing using a co‐design approach and the barriers and facilitators to implementing social prescribing. The institute provided networking opportunities and the sharing of resources relevant to social prescribing. It also enabled participants to identify additional opportunities for addressing gaps, including funding and policy. The findings contribute to how convening of national stakeholders could be applied to other areas beyond social prescribing, given our understanding of the complex interplay between the macro‐ and micro‐level settings in which interventions are deployed.

## Author Contributions

**Marianne Saragosa:** conceptualization, data curation, formal analysis, investigation, project administration, writing–original draft, writing–review and editing. **Kate Mulligan:** funding acquisition, project administration, writing–review and editing. **Sonia Hsiung:** funding acquisition, writing–review and editing. **Srija Biswas:** funding, writing–review and editing. **Kiffer Card:** writing–review and editing. **Paul C. Hébert:** writing–review and editing. **Vivian Welch:** writing–review and editing. **Michelle L.A. Nelson:** conceptualization, writing–review and editing.

## Ethics Statement

The Mount Sinai Hospital Research Ethics Board granted ethical approval.

## Consent

All participants provided written informed consent before being involved in semi‐structured interviews.

## Conflicts of Interest

The authors declare no conflicts of interest.

## Supporting information

Supporting information.

## Data Availability

Research data are not shared.
