# Supplementary material for: A Qualitative Study of National Perspectives on Advancing Social Prescribing Using Co‐Design in Canada
Source: Health Expect. 2024 Jul 10;27(4):e14144. doi: 10.1111/hex.14144 (PMC11234137; doi:10.1111/hex.14144)
Supplement: Supplementary file 1 — Supporting information. [file HEX-27-e14144-s001.docx]

**CISP Appendix H – Field notes template**

**Event:**

**Actors (e.g., roles):**

**Date/time:**

**Location:**

**Length of observation:**

| **Summary of Activities**  Write a summary of the observed event or activity. | *MEMOS: Notes to self—things to follow up on or highlight—e.g., notable analysis, contacts, sources, follow-up questions* |
| --- | --- |
| **Narrative/Interpretation of Observations**  Pay attention to:  What people are doing, actual words and phrases people use  Be specific as possible, avoid generalizing  Avoid projecting internal states and emotion |  |
